# Polymorphisms in RAS/RAF/MEK/ERK Pathway Are Associated with Gastric Cancer

**DOI:** 10.3390/genes10010020

**Published:** 2018-12-28

**Authors:** Patricio Gonzalez-Hormazabal, Maher Musleh, Marco Bustamante, Juan Stambuk, Raul Pisano, Hector Valladares, Enrique Lanzarini, Hector Chiong, Jorge Rojas, Jose Suazo, V. Gonzalo Castro, Lilian Jara, Zoltan Berger

**Affiliations:** 1 Human Genetics Program, Institute of Biomedical Sciences (ICBM), School of Medicine, University of Chile, Santiago 8380453, Chile; vgcastro@gmail.com (V.G.C.); ljara@uchile.cl (L.J.); 2 Department of Surgery, University of Chile Clinical Hospital, Santiago 8380456, Chile; mmuslehk@gmail.com (M.M.); hvalladares@hcuch.cl (H.V.); elanzarini@hotmail.com (E.L.); jrojascaro@yahoo.com (J.R.); 3 Department of Surgery, School of Medicine at Eastern Campus, University of Chile, Santiago 7500922, Chile; mbustama@med.uchile.cl; 4 Department of Surgery, San Juan de Dios Hospital, Santiago 8350488, Chile; juanstambuk@gmail.com (J.S.); raul.pisano@redsalud.gov.cl (R.P.); 5 Department of Surgery, Barros Luco Hospital, Santiago 8900085, Chile; drhectorchiong@gmail.com; 6 Institute for Research in Dental Sciences, School of Dentistry, University of Chile, Santiago 8380492, Chile; jsuazo@odontologia.uchile.cl; 7 Department of Gastroenterology, University of Chile Clinical Hospital, Santiago 8380456, Chile; berger.zoltan@gmail.com

**Keywords:** gastric cancer, polymorphism, RAS/RAF/MEK/ERK pathway, MAPK pathway, association study

## Abstract

The RAS/RAF/MEK/ERK pathway regulates certain cellular functions, including cell proliferation, differentiation, survival, and apoptosis. Dysregulation of this pathway leads to the occurrence and progression of cancers mainly by somatic mutations. This study aimed to assess if polymorphisms of the RAS/RAF/MEK/ERK pathway are associated with gastric cancer. A case-control study of 242 gastric cancer patients and 242 controls was performed to assess the association of 27 single nucleotide polymorphisms (SNPs) in the RAS/RAF/MEK/ERK pathway genes with gastric cancer. Analyses performed under the additive model (allele) showed four significantly associated SNPs: *RAF1* rs3729931 (Odds ratio (OR) = 1.54, 95%, confidence interval (CI): 1.20–1.98, *p*-value = 7.95 × 10^−4^), *HRAS* rs45604736 (OR = 1.60, 95% CI: 1.16–2.22, *p*-value = 4.68 × 10^−3^), *MAPK1* rs2283792 (OR = 1.45, 95% CI: 1.12–1.87, *p*-value = 4.91 × 10^−3^), and *MAPK1* rs9610417 (OR = 0.60, 95% CI: 0.42–0.87, *p*-value = 6.64 × 10^−3^). Functional annotation suggested that those variants or their proxy variants may have a functional effect. In conclusion, this study suggests that *RAF1* rs3729931, *HRAS* rs45604736, *MAPK1* rs2283792, and *MAPK1* rs9610417 are associated with gastric cancer.

## 1. Introduction

The latest data from CANCER TODAY (part of the GLOBOCAN project) [1] confirm that gastric cancer is the fifth most common cancer and the third leading cause of cancer-related mortality. Incidence rates of gastric cancer vary dramatically according to the region of the world. The age standardized rate (ASR) of incidence is 22.4/100,000 in East Asia, 11.4/100,000 in Eastern Europe, and 9.5/100,000 in South America, as compared to less than 5/100,000 in Africa, Australia, North America, and Northern Europe. Chile has the seventh highest incidence of gastric cancer, with an ASR of 17.8/100,000, similar to Latin American countries of Central America and the Pacific coast of South America, such as Peru, Guatemala, Ecuador, and Costa Rica. Risk factors for gastric cancer include high salt intake, low fruit and vegetable consumption, smoking, *Helicobacter pylori* infection, and genetic factors [2]. In 2015, Mocellin et al. [3] published the results of a systematic review and meta-analysis of the evidence linking polymorphisms with gastric cancer, and found 11 variants that are significantly associated with a high level of summary evidence. Some of the polymorphisms were found in hypothesis-driven studies based on a candidate gene approach. In this study, Asian ancestry associated with some polymorphisms, and Caucasian ancestry with other polymorphisms.

The mitogen-activated protein kinase (MAPK) pathway, also known as the RAS/RAF/MEK/ERK pathway, is a signaling cascade that regulates certain cellular functions in physiological conditions, including cell proliferation, differentiation, survival, and apoptosis [4]. Malfunction in MAPK signaling leads to the occurrence and progression of cancers, mainly by somatic mutations. In fact, RAS has been recognized as an oncogene widely activated by mutation in all cancers. *KRAS* is the most frequently mutated gene (20%), followed by *NRAS* (8%), and *HRAS* (3.3%) [4]. The Cancer Genome Atlas Research Network [5] and the Asian Cancer Research Group [6] found activating mutations in *KRAS* to be present in gastric cancer tumors, particularly in 25% of microsatellite-instable (MSI) subtypes of gastric cancer. On the other hand, gastrin mediates its own actions by up-regulating phosphorylation of extracellular regulated kinase (ERK) through the MAPK pathway. Gastrin is a peptide involved in secretion of gastric acid and growth of the gastrointestinal tract, and is capable of stimulating growth in gastric cancer cell lines [7].

The RAS/RAF/MEK/ERK pathway is triggered by the activation of tyrosine kinase receptors (RTK), G protein-coupled receptors (GCPR), and integrins [8,9,10]. More than 50 RTKs have been described [8], of which EGFR, HER2/ErrB2, HER3/ErrB3, PDGFR-β, FGFR2, AKT, and c-MET have been described as upregulated in gastric cancer [5,11,12,13,14,15]. Following ligand binding, RTKs dimerize and transactivate by tyrosine-phosphorylation to provide docking sites for growth factor receptor-bound protein 2 (Grb2). Grb2 acts as an adaptor protein that recruits SHC-transforming protein (Shc) and Son of Sevenless (SOS) near to the plasma membrane. SOS activates the GTPase activity of Ras—a family of proteins attached to the plasma membrane [16]. Ras proteins activate RAF proto-oncogene serine/threonine-protein kinase (c-Raf), which phosphorylates mitogen-activated protein kinase kinase (MEK). The last step of the cascade is activation of extracellular signal-regulated kinase (ERK). Activated ERK can enter the nucleus to phosphorylate over 250 cellular substrates involved in migration, proliferation, and survival, among other functions [17].

The aim of this study was to find associations between genetic variants in crucial genes of the (RTK)/RAS/RAF/MEK/ERK pathway and gastric cancer. In a hospital-based, case-control study of Chilean subjects, we assessed the association of 27 single nucleotide polymorphisms (SNPs) belonging to the RAS/RAF/MEK/ERK genes with gastric cancer.

## 2. Materials and Methods

### 2.1. Subjects

A total of 242 individuals (169 men and 73 women) with a mean age of 64.6 ± 11.7 (range = 25 to 88) years with confirmed histopathological diagnosis of gastric adenocarcinoma were recruited at the time of surgical resection between December 2010 and August 2017 from four different hospitals in Santiago de Chile: University of Chile Clinical Hospital and Biobanco de Tejidos y Fluidos de la Universidad de Chile (BTUCH), Salvador Hospital, Barros Luco Trudeau Hospital, and San Juan de Dios Hospital. In all cases, the tumor was located distally to the cardia. Clinicopathological features of included patients are shown in Table 1. Tumor size, depth of invasion, and lymph node metastasis were obtained from the histopathological report. Lauren’s criteria were used to classify tumors as intestinal or diffuse.

Blood samples from 242 controls (126 men and 116 women) with a mean age of 45.5 ± 16.3 (range = 20 to 82) years were obtained from individuals who underwent endoscopy, requested by their physician, at the Department of Gastroenterology at the University of Chile Clinical Hospital. Those with evidence of peptic or duodenal ulcer or endoscopic evidence suggestive of premalignant lesions (such as atrophic gastritis or intestinal metaplasia) were not included. 

This study was approved by the ethical committees of the following institutions: University of Chile School of Medicine (#045/2015), University of Chile Clinical Hospital (#078/2015), Metropolitan South-Santiago Public Health Agency (#MK523B-118), Metropolitan East-Santiago Public Health Agency (#24/01/2012), and Metropolitan West-Santiago Public Health Agency (#236/2009). All participants gave their written informed consent. The study was performed in accordance with the Declaration of Helsinki.

### 2.2. Genotyping and SNP Selection

Blood samples were collected in EDTA Vacutainers. Genomic DNA was isolated via the salting out method and Proteinase K, or according to the method described by Chomczynski and Sacchi [18]. In both cases, genomic DNA was further purified using Monarch PCR and DNA cleanup columns (New England Biolabs (NEB), Ipswich, MA, USA). Genotyping was performed using an Infinium Global Screening Array-24 BeadChip (Illumina, San Diego, CA, USA) according to the manufacturer’s instructions, at the Human Genomics Facility (HuGe-F) in Erasmus MC, Netherlands. Per-individual and per-marker quality controls were conducted according to the guidelines in Anderson et al. [19] using PLINK 1.9 (www.cog-genomics.org/plink/1.9/). All the studied patients and studied SNPs passed the quality control test.

Studied genes were selected from the (RTK)/RAS/RAF/MEK/ERK pathway described in KEGG pathway “gastric cancer” (map05226). The pathway includes the following proteins (and the corresponding encoding genes): EGFR (*EGFR*), HER2 (*ERBB2*), Shc (*SHC1*), Grb2 (*GRB2*), SOS (*SOS1*, *SOS2*), Ras (*HRAS*, *KRAS*, *NRAS*), Raf (*ARAF*, *BRAF*, *RAF1*), MEK (*MAP2K1*, *MAP2K2*), and ERK1/2 (*MAPK1*, *MAPK3*). In addition to *EGFR* and *ERBB2*, we also included other tyrosine-kinase receptors that are known to trigger the RAS/RAF/MEK/ERK pathway and that are upregulated in gastric cancer: c-MET (*MET*), FGFR2 (*FGFR2*), HER3 (*ERBB3*), and PDGFR-β (*PDGFRB*). From the GSA array, the following criteria were used to select the SNPs for analysis: (1) located between 5 kb upstream from the transcription start site and 5 kb downstream from the stop triplet according to the GRCh37 assembly of the human genome; (2) minor allele frequency (MAF) higher than 0.10; (3) not in linkage disequilibrium (LD) (r^2^ > 0.8) with other SNPs contained in the array; (4) with no departure from Hardy–Weinberg equilibrium (HWE) in the studied population (*p* < 0.01); (5) located in autosomes; and (6) have regulomeDB score [20] 1 or 2 (themselves or their proxy SNPs) according to Ad Mixed American (AMR) population data retrieved with LDlink 3.2.0 [21] from the 1000 Genomes Project Phase 3 (Version 5) data. No SNPs fulfilled all the criteria for the *BRAF*, *SOS2*, *NRAS*, *ARAF*, and *MAPK3* genes. Table S1 describes the list of 27 SNPs analyzed in this study.

### 2.3. Functional Annotation

RegulomeDB score [20] was used to predict whether a non-coding SNP affects regulatory function by means of information regarding epigenetic signatures related to gene expression (enhancer histone marks, promoter histone marks, DNAse hypersensitive sites), and protein-binding sites (Chromatin ImmunoChIP seq). Association of an SNP with gene expression (expression Quantitative-Trait Loci) was assessed using the Genotype-Tissue Expression (GTEx) project [22].

### 2.4. Statistical Analyses

Manipulation of the set of genotypes and statistical analyses were performed using PLINK 1.9 [23]. The exact test was used to detect departures from the HWE. A logistic regression analysis was performed to assess association of SNPs under the allele (additive), dominant, co-dominant, and recessive models. Fisher’s exact test of independence was used to compare genotype distribution between cases and controls. To infer population stratification, we used the set of genotypes obtained from Infinium Global Screening Array (Illumina, CA, USA) excluding—pruning—SNPs from extended regions of high LD (r^2^ > 0.2) using --indep–pairwise in PLINK 1.9. A final set of 184,909 autosome SNPs were submitted for principal component analysis (PCA) using --pca in PLINK 1.9. Principal component (PC)1 and PC2 were included in the logistic regression analyses as covariates to obtain *p*-values adjusted for population stratification [24]. False discovery rate (FDR) was used to correct for multiple testing according to the Benjamini–Hochberg procedure [25]. The cutoff of the FDR adjusted *p*-value (*q*-value) was 0.05. We computed the power for genetic association under the allele model using Quanto 1.2.4. (http://biostats.usc.edu/Quanto.html).

## 3. Results

The genotype distributions of SNPs selected for this study among gastric cancer cases, intestinal-type gastric cancer cases, diffuse-type gastric cancer cases, and controls are shown in Table S2. Analyses performed under the additive model (allele) showed four significantly associated SNPs (Table 2): *RAF1* rs3729931 (*p*-value = 7.95 × 10^−4^, *q*-value = 0.018), *HRAS* rs45604736 (*p*-value = 4.68 × 10^−3^, *q*-value = 0.036), *MAPK1* rs2283792 (*p*-value = 4.91 × 10^−3^, *q*-value = 0.036), and *MAPK1* rs9610417 (*p*-value = 6.64 × 10^−3^, *q*-value = 0.037). Results were comparable when a logistic regression analysis was performed adjusting for PC1 and PC2 as measures of population stratification (Table 2). The polymorphism *MAPK1* rs743409 showed significant association with gastric cancer in the additive model (crude *p*-value = 0.036, *p*-value = 0.033 adjusted for PC1 and PC2) but was not considered for further analysis after adjusting for multiple comparisons (crude *q*-value = 0.157, *q*-value = 0.145, adjusted for PC1 and PC2). We then evaluated the risk conferred by the associated allele according to the genotype (Table 3). For *RAF1* rs3729931 the associated allele (T) acts in a recessive manner to confer risk (TT versus TC + CC OR = 1.98, 95% CI 1.30–3.04). Nevertheless, *HRAS* rs45604736 (CC + CT versus TT OR = 1.82, 95% CI 1.24–2.66) and *MAPK1* rs2283792 (TT + TG versus GG OR = 1.77, 95% CI 1.20–2.61), both act in a dominant way. The minor allele *MAPK1* rs9610417 T is associated with gastric cancer as a protector allele under the dominant model (OR = 0.56, 95% CI 0.38–0.84).

A stratified analysis was conducted for the following clinicopathological features: the two main histological subtypes (intestinal and diffuse), tumor size, and TNM stage. Table S3 shows the results of the association study under the additive model. There are no differences in the distribution of genotype frequencies between both strata of each clinicopathological feature.

Considering the sample size used in the present study, the statistical power (1-beta) to estimate the obtained effect size of each associated SNP in the additive model was: beta = 0.09 for rs3729931, beta = 0.19 for rs45604736, beta = 0.19 for rs2283792, and beta = 0.18 for rs9610417, all under the accepted threshold of 0.2.

Table S4 summarizes functional annotation for the four significantly associated SNPs: *RAF1* rs3729931, *HRAS* rs45604736, *MAPK1* rs2283792, and *MAPK1* rs9610417. *RAF1* rs3729931 is located in intron 15 of *RAF1* gene (NM_002880.3: c.1669-36C>T). According to data from GTEx, allele T is associated with low expression of the *RAF1* gene, and therefore is considered as an expression quantitative trait locus (eQTL). *HRAS* rs45604736 corresponds to a polymorphism in the promoter region of *HRAS* gene (−1115T>C). Functional annotation of epigenetic signatures gives strong evidence that this variant lies in a region of transcriptionally active chromatin. Nevertheless, searches in the GTEx database give no results. Non-coding associated variants from genome-wide association studies (GWAS) are enriched in enhancer and DNAse hypersensitive sites [26,27]; therefore, an SNP in these sites is likely to be functional. *MAPK1* rs2283792 (c.857-3854A>C) is located in intron 6 of the *MAPK1* gene and belongs to an extensive LD block spanning 128 kb of the *MAPK1* gene, comprising 37 SNPs according to AMR population data retrieved with LDlink 3.2.0 [21] from the 1000 Genomes Project Phase 3 (Version 5) data. The SNP rs2283792 allele T increases the expression of *MAPK1* in the esophagus (muscularis) according to GTEx, as well as for 33 of the 36 SNPs that are in LD with this variant. The analysis of epigenetic signatures and protein binding-sites suggests that rs5999521 and rs3788332 could have biological effects explaining the association of rs2283792. Experimental assessment of the effects of rs5999521 and rs3788332 on *MAPK1* expression is needed to support this conclusion. *MAPK1* rs9610417 corresponds to a substitution in intron 1 of *MAPK1* (c.120-28740G > A). Data regarding the AMR population in the 1000 Genomes Project Phase 3 (Version 5) reveals that this SNP is in LD with 20 SNPs in a block of 115 kb (Table S3). Eighteen of them are eQTL in skeletal muscle, where rs9610417 allele T is associated with low MAPK1 expression. This is in accordance with the finding that this allele associates as a protective allele in the present study. Five proxy SNPs—rs9607272, rs9610496, rs9610470, rs9610487, and rs9610504—of rs9610417 have RegulomeDB scores, which suggests a functional effect. Therefore, those SNPs could account for the association found with rs9610417. Functional in vitro experiments to test this association would aid to prove this link.

## 4. Discussion

In the present case-control study, we aimed to assess the association between SNPs in genes in the RAS/RAF/MEK/ERK pathway and gastric cancer, based on 242 cases of gastric cancer and in 242 controls from Chile. We found significant association of the SNPs *RAF1* rs3729931, *HRAS* rs45604736, and *MAPK1* rs2283792 and rs9610417 with gastric cancer. To the best of our knowledge, this is the first study investigating the association between SNPs in the RAS/RAF/MEK/ERK pathway and gastric cancer.

*RAF1* rs3729931 was found to be associated with cardiac hypertrophy in a GWAS conducted among patients from an Amish population [28]. Pathogenic gain-of-function variants in *RAF1*—among other genes including *PTPN11*, *SOS1*, and *KRAS*—cause Noonan syndrome. Congenital heart defects are a life-threatening part of this syndrome and shorten the life expectancy of patients. On the other hand, patients with “RASopathies”—syndromes arising from gain-of-function variants in genes from the Ras signaling pathway—are cancer-prone, that is, they are at high risk for neoplasms [29]. According to the GTEx project [22], the rs3729931 T allele is an eQTL, which significantly associated with low expression of *RAF1* in human transformed fibroblast (single tissue *p*-value 1.3 × 10^−7^). Nevertheless, allele rs3729931 T is also associated with increased *RAF1* expression in other tissues, such as esophagus mucosa (single tissue *p*-value 8.3 × 10^−4^) and breast (single tissue *p*-value 4.8 × 10^−4^), albeit these associations were not statistically significant after multi-tissue correction (m-value). Interestingly, in almost all tissues, rs3729931 T is associated with increased expression of *MKRN2*, a gene that partially overlaps with *RAF1* in the opposite DNA strand. *MKRN2* encodes Makorin RING zinc finger-2 protein, a novel ubiquitin E3 ligase with no target proteins defined yet, except for NF-kB [30]. Recently, Jiang et al. [31] demonstrated that this protein inhibits progression in non-small cell lung cancer cells. Remarkably, according to The Cancer Genome Atlas (TCGA) data, this gene is underexpressed in lung adenocarcinoma (*p*-value = 1.59 × 10^−3^) but overexpressed in gastric cancer tissue (*p*-value = 1.03 × 10^−2^) (data retrieved from Reference [32]).

The SNP rs45604736 corresponds to a polymorphism in the promoter region of *HRAS* gene (−1115T > C). This gene encodes one of the three Ras small GTPases (HRAS, KRAS, and NRAS). Somatic mutations in *KRAS* are common in gastric cancer tumors [5,33], *NRAS* is frequently mutated in melanoma, and *HRAS* to some extent in breast cancer [16]. Not much is known about the role of *HRAS* in gastric cancer. This gene is overexpressed in gastric carcinoma in samples from TCGA (*p*-value = 0.004, data retrieved from Reference [32]). Wu et al. [34] demonstrated experimentally that overexpression of this gene in gastric cancer cell lines promotes proliferation, metastasis, and angiogenesis, and is possibly mediated by PI3K-AKT and Raf-1 pathways.

Two SNPs of *MAPK1*—rs2283792 and rs9610417—were demonstrated to be associated with gastric cancer. Both SNPs are associated with expression levels of *MAPK1* gene, which agrees with its size effect found in this study. *MAPK1* encodes Erk2, which is 84% identical to Erk1 (encoded by *MAPK3*) and with a functionally redundant role [35]. Notably, *MAPK1* is significantly overexpressed in gastric adenocarcinoma (*p*-value < 0.001) but *MAPK3* is underexpressed (*p*-value = 0.001) (TCGA data retrieved from Reference [32]). Both kinases are activated by phosphorylation by MEK to regulate a number of cellular events, including cell proliferation and survival [36]. In a previous GWAS, this variant was found to be associated in subjects with multiple sclerosis. ERK signaling is pleiotropic and plays a crucial role in regulating immune cell function and as such has been implicated in autoimmune disorders, such as multiple sclerosis [37].

The effect size reported in the present article is comparable to those obtained with a high level of summary evidence, as in a meta-analysis by Mocellin et al. [3]. For example, among SNPs associated with increasing risk of gastric cancer (GC), *PLCE1* rs2274223 has an OR of 1.57, and *PSCA* rs2294008 an OR of 1.33. Regarding the protective SNPs, *MUC1* rs2070803 has the lowest OR (OR = 0.59).

Studies addressing the association of polymorphisms in the RTK/RAS/RAF/MEK/ERK pathway with gastric cancer are scarce. Zhang et al. [38] found that *HRAS* rs12628 (or T81C) was significantly associated with GC under the dominant model (OR = 3.65, 95% CI = 2.22–6.00) in a Chinese population. To the best of our knowledge, no other studies have been published studying the (RTK)/RAS/RAF/MEK/ERK pathway polymorphisms and their association with GC risk.

Some limitations need to be considered. First, this is a multi-centric hospital-based study that does not necessarily represent the general population of Santiago, Chile. Second, information regarding environmental risk factors was not available, which did not allow us to assess gene–environment interactions. Third, our findings were based on unadjusted estimations of effect sizes. According to Pirinen et al. [39], when disease prevalence is lower than 2% (as in the case of gastric cancer), adjustment for known covariates, such as age and sex, can substantially reduce the statistical power. Nevertheless, we adjusted for population stratification, which is actually a confounder correlated with both allele frequency and the disease. Fourth, even though the sample size was large enough to reach the desired statistical power for the additive model, our findings must be confirmed in a replication study.

In conclusion, using a case-control approach we found that rs3729931 (*RAF1*), rs45604736 (*HRAS*), rs2283792, and rs9610417 (*MAPK1*) are associated with gastric cancer. Our study provides new insights in the contribution of polymorphisms in the RAS/RAF/MEK/ERK pathway to cancer risk. Furthermore, larger studies in other populations are needed to confirm our findings.

## Figures and Tables

**Table 1:** Clinicopathological characteristics of gastric cancer patients.

Variable	Gastric Cancer (%)
TNM 8th edition stage	
IA	34 (14.0%)
IB	19 (7.9%)
IIA	24 (9.9%)
IIB	0 (0%)
IIIA	0 (0%)
IIIB	149 (61.6%)
IIIC	1 (0.4%)
IV	12 (1.2%)
Not available	3 (1.2%)
Lauren’s classification	
Intestinal	132 (54.5%)
Diffuse/Mixed	109 (45.1%)
Indeterminate	1 (0.4%)
Tumor size	
<5 cm	112 (46.3%)
≥5 cm	129 (53.3%)
Not available	1 (0.4%)

**Table 2:** Single nucleotide polymorphisms (SNPs) associated with gastric cancer with the smallest *p*-value for allele model.

rsID	Gene	Minor Allele	OR (95% CI) (1)	*p*-Value (1)	FDR *q*-Value (1)	OR (95% CI) (2)	*p*-Value (2)	FDR *q*-Value (3)	OR (95% CI) (3)	*p*-Value (3)	FDR *q*-Value (3)
rs3729931	*RAF1*	T	1.54 (1.20–1.98)	7.95 × 10^−4^	0.018	1.52 (1.18–1.96)	1.29 × 10^−3^	0.028	1.52 (1.13–2.0)	4.95 × 10^−3^	0.067
rs45604736	*HRAS*	C	1.60 (1.16–2.22)	4.68 × 10^−3^	0.036	1.58 (1.14–2.20)	5.24 × 10^−3^	0.036	1.45 (1.01–2.11)	4.89 × 10^−2^	0.189
rs2283792	*MAPK1*	T	1.45 (1.12–1.87)	4.91 × 10^−3^	0.036	1.46 (1.13–1.90)	5.23 × 10^−3^	0.036	1.48 (1.10–2.00)	9.44 × 10^−3^	0.085
rs9610417	*MAPK1*	T	0.60 (0.42–0.87)	6.64 × 10^−3^	0.037	0.59 (0.41–0.86)	6.55 × 10^−3^	0.036	0.54 (0.35–0.82)	3.89 × 10^−3^	0.067

(1) unadjusted, (2) adjusted by principal component (PC)1 and PC2 (242 cases and 242 controls), (3) adjusted by age, sex, PC1 and PC2 (242 cases and 242 controls). Minor allele is the effect allele. OR: Odds ratio; FDR: false discovery rate.

**Table 3:** Association of rs3729931, rs45604736, rs2283792, rs9610417 genotypes with gastric cancer.

	rs3729931 (*RAF1*)	*p*-Value (1)	rs45604736 (*HRAS*)	*p*-Value (1)	rs2283792 (*MAPK1*)	*p*-Value (1)	rs9610417 (*MAPK1*)	*p*-Value (1)
aa + aA vs. AA	1.63 (1.10–2.43)	1.61 × 10^−2^	1.82 (1.24–2.66)	2.20 × 10^−3^	1.77 (1.20–2.61)	3.93 × 10^−3^	0.56 (0.38–0.84)	4.53 × 10^−3^
aa vs. aA + AA	1.98 (1.30–3.04)	1.65 × 10^−3^	1.41 (0.56–3.57)	4.67 × 10^−1^	1.46 (0.93–2.30)	1.55 × 10^−1^	0.67 (0.19–2.39)	5.34 × 10^−1^
aa vs. AA	2.39 (1.44–3.96)	7.04 × 10^−4^	1.71 (0.66–4.35)	2.64 × 10^−1^	2.00 (1.19–3.37)	8.89 × 10^−3^	0.57 (0.16–2.05)	3.86 × 10^−1^
aA vs. AA	1.35 (0.88–2.07)	1.70 × 10^−1^	1.83 (1.23–2.73)	2.92 × 10^−3^	1.69 (1.12–2.55)	1.28 × 10^−2^	0.56 (0.37–0.84)	5.62 × 10^−3^

(1) unadjusted.

## Supplementary Materials

The following are available online at http://www.mdpi.com/2073-4425/10/1/20/s1. Table S1: Description of the 27 SNPs analyzed in this study, Table S2: Genotype frequencies of studied SNPs among gastric cancer cases and controls, Table S3: Association for the allele model according to clinicopathological features, Table S4: Proxy SNPs of associated variants and their functional annotation.
