# Supplementary material for: Polymorphisms in RAS/RAF/MEK/ERK Pathway Are Associated with Gastric Cancer
Source: Genes (Basel). 2018 Dec 28;10(1):20. doi: 10.3390/genes10010020 (PMC6356706; doi:10.3390/genes10010020)
Supplement: Supplementary file 1 [file genes-10-00020-s001.zip › table_S1.pdf]

Table S1. Description of the 27 SNPs analyzed in this study.

rsID	Gene	HGVS	[1]	[2]	Allele frequency [3]					[4]
					AFR	AMR	EAS	EUR	SAS	
rs10184015	*SOS1*	NM_005633.3: c.1859-1142T>C	G/A	A	0.57	0.17	0.19	0.09	0.19	0.12
rs2290159	*RAF1*	NM_002880.3:c.1417+170C>G	G/C	C	0.32	0.19	0.03	0.22	0.09	0.20
rs3729931	*RAF1*	NM_002880.3:c.1669-36C>T	C/T	T	0.65	0.47	0.06	0.38	0.21	0.42
rs73812837	*RAF1*	NM_002880.3:c.-26-2203C>T	G/A	A	0.25	0.13	0.03	0.11	0.07	0.13
rs10228436	*EGFR*	NM_005228.4:c.1881-600G>A	G/A	A	0.22	0.39	0.54	0.35	0.44	0.43
rs11514996	*EGFR*	NM_005228.4:c.2283+1296C>T	C/T	T	0.15	0.40	0.62	0.35	0.41	0.40
rs11770506	*EGFR*	NM_005228.4:c.88+3321T>C	T/C	C	0.13	0.41	0.68	0.35	0.40	0.47
rs17172438	*EGFR*	NM_005228.4:c.89-58442T>C	T/C	C	0.36	0.16	0.03	0.18	0.08	0.13
rs2740761	*EGFR*	NM_005228.4:c.2470-3426C>T	C/T	T	0.21	0.18	0.08	0.23	0.09	0.23
rs6593201	*EGFR*	NM_005228.4:c.88+37628A>G	A/G	G	0.91	0.89	0.98	0.81	0.83	0.18
rs712829	*EGFR*	NM_005228.4:c.-216G>T	G/T	T	0.25	0.21	0.06	0.31	0.30	0.23
rs7795743	*EGFR*	NM_005228.4:c.2469+959G>A	A/G	G	0.57	0.57	0.18	0.61	0.45	0.35
rs45604736	*HRAS*	NM_001130442.2:c.-1115T>C	T/C	C	0.00	0.12	0.15	0.03	0.04	0.16
rs9266	*KRAS*	NM_033360.3:c.*633T>C	C/T	T	0.28	0.51	0.82	0.52	0.69	0.51
rs1347069	*MAP2K1*	NM_002755.3:c.81-996C>T	G/A	A	0.07	0.28	0.13	0.25	0.21	0.34
rs62010232	*MAP2K1*	NM_002755.3:c.569-16806A>G	G/A	A	0.05	0.12	0.06	0.13	0.07	0.11
rs959260	*GRB2*	NM_002086.4:c.78+20210G>A	T/C	C	0.83	0.26	0.11	0.17	0.25	0.13
rs350912	*MAP2K2*	NM_030662.3:c.919+423T>C	C/T	T	0.15	0.16	0.08	0.30	0.31	0.21
rs1823059	*MAP2K2*	NM_030662.3:c.303+1424C>T	C/T	T	0.07	0.24	0.26	0.23	0.31	0.20
rs2283792	*MAPK1*	NM_002745.4:c.857-3854A>C	G/T	T	0.36	0.38	0.56	0.48	0.54	0.39
rs4821401	*MAPK1*	NM_002745.4:c.119+7040A>G	T/C	C	0.00	0.17	0.21	0.03	0.12	0.17
rs743409	*MAPK1*	NM_002745.4:c.857-1944T>C	C/T	T	0.36	0.34	0.54	0.48	0.49	0.33
rs9340	*MAPK1*	NM_002745.4:c.*3186C>T	C/T	T	0.35	0.38	0.20	0.44	0.31	0.39
rs9610417	*MAPK1*	NM_002745.4:c.119+21642G>A	C/T	T	0.08	0.18	0.09	0.25	0.10	0.19
rs1017375	*PDGFRB*	NM_001355016.1:c.*805C>T	G/A	A	0.22	0.11	0.01	0.05	0.06	0.14
rs10066011	*PDGFRB*	NM_001355016.1:c.-152-8335A>G	T/C	C	0.17	0.14	0.26	0.1	0.21	0.15
rs58746386	*PDGFRB*	NM_001355016.1:c.-153+4691A>G	T/C	C	0.16	0.17	0.13	0.2	0.12	0.18

HSVG: Human Genome Variation Society nomenclature, [1] mayor allele/minor allele defined according to GRCh37 human genome assembly, [2] assessed allele, [3] data from 1000 genomes phase 3, [4] allele frequency in controls, present study.
