# Supplementary material for: Polymorphisms in RAS/RAF/MEK/ERK Pathway Are Associated with Gastric Cancer
Source: Genes (Basel). 2018 Dec 28;10(1):20. doi: 10.3390/genes10010020 (PMC6356706; doi:10.3390/genes10010020)
Supplement: Supplementary file 1 [file genes-10-00020-s001.zip › table_S2.pdf]

Table S2. Genotype frequencies of studied SNPs among gastric cancer cases and controls.

rsID	Gene	Genotype frequencies [1]				*p*-value [2]	*p*-value [3]	*p*-value [4]	HWE
		Gastric Cancer	Intestinal-type	Diffuse-type	Controls				
rs10184015	*SOS1*	4/50/188	2/26/104	2/23/84	7/42/193	0.45	0.65	0.69	0.03
rs2290159	*RAF1*	19/77/145	10/47/75	9/30/69	12/70/159	0.29	0.18	0.47	0.22
rs3729931	*RAF1*	74/111/57	40/56/36	34/54/21	44/117/81	0.00	0.03	0.00	0.90
rs73812837	*RAF1*	10/54/176	4/35/92	6/19/83	7/48/187	0.56	0.29	0.43	0.08
rs10228436	*EGFR*	41/116/84	17/61/53	24/54/31	43/121/77	0.80	0.20	0.61	0.79
rs11514996	*EGFR*	38/115/86	20/63/47	18/52/38	52/123/67	0.09	0.16	0.32	0.80
rs11770506	*EGFR*	5/51/185	2/25/104	3/26/80	6/53/183	0.94	0.73	0.86	0.40
rs17172438	*EGFR*	32/113/95	13/61/57	19/51/38	41/109/92	0.55	0.16	0.87	0.42
rs2740761	*EGFR*	10/74/157	4/34/93	6/40/63	17/75/150	0.38	0.13	0.55	0.10
rs6593201	*EGFR*	5/61/175	3/29/99	2/31/76	4/79/159	0.19	0.08	0.71	0.13
rs712829	*EGFR*	23/85/125	11/43/72	12/41/53	14/80/142	0.19	0.58	0.10	0.58
rs7795743	*EGFR*	24/115/101	13/60/58	11/55/42	25/119/98	0.95	0.78	0.97	0.26
rs45604736	*HRAS*	11/87/141	4/52/75	7/35/65	8/59/175	0.01	0.01	0.07	0.32
rs9266	*KRAS*	55/119/67	28/70/34	27/49/32	69/110/63	0.36	0.26	0.72	0.16
rs1347069	*MAP2K1*	22/100/119	16/60/56	6/39/63	29/106/106	0.39	0.96	0.03	0.77
rs62010232	*MAP2K1*	8/46/183	4/22/103	4/24/79	6/38/186	0.64	0.91	0.31	0.03
rs959260	*GRB2*	10/51/181	4/26/102	6/24/79	4/57/180	0.24	0.50	0.16	1.00
rs1823059	*MAP2K2*	8/85/148	4/48/80	4/37/67	7/83/152	0.92	0.93	0.90	0.32
rs350912	*MAP2K2*	11/78/150	7/40/83	4/37/67	8/85/149	0.67	0.46	1.00	0.44
rs2283792	*MAPK1*	54/125/62	25/75/32	29/49/30	40/110/92	0.01	0.02	0.05	0.19
rs4821401	*MAPK1*	5/53/183	2/30/100	3/22/83	5/72/165	0.14	0.28	0.20	0.49
rs743409	*MAPK1*	35/120/86	18/66/48	17/53/38	25/109/107	0.11	0.28	0.17	0.77
rs9340	*MAPK1*	24/115/103	13/63/56	11/52/46	38/114/90	0.13	0.26	0.35	0.89
rs9610417	*MAPK1*	4/52/180	2/29/99	2/23/80	6/79/153	0.01	0.06	0.08	0.40
rs10066011	*PDGFRB*	6/60/175	3/32/96	3/28/78	4/62/176	0.85	0.86	0.84	0.80
rs1017375	*PDGFRB*	7/53/181	5/33/93	2/20/87	6/53/183	0.98	0.52	0.73	0.40
rs58746386	*PDGFRB*	11/66/164	4/37/90	7/29/73	4/80/158	0.10	0.44	0.04	0.13

[1] aa/Aa/AA, being A the mayor allele and a the minor allele. [2] Gastric cancer versus controls, Fisher's exact test of independence.
[3] Intestinal-type versus controls, Fisher's exact test of independence. [4] Diffuse-type versus controls, Fisher's exact test of independence.
HWE: Exact test p-value for departures from Hardy-Weinberg Equilibrium.
