# Supplementary material for: Polymorphisms in RAS/RAF/MEK/ERK Pathway Are Associated with Gastric Cancer
Source: Genes (Basel). 2018 Dec 28;10(1):20. doi: 10.3390/genes10010020 (PMC6356706; doi:10.3390/genes10010020)
Supplement: Supplementary file 1 [file genes-10-00020-s001.zip › table_S3.pdf]

Table S3. Association for the allele model according to clinicopathological features.

a) Lauren's classification

rsID	Gene	Minor allele	Intestinal-type OR (95% CI)	*p*-value (unadjusted)	Diffuse-type OR(95%CI)	*p*-value (unadjusted)	*p*-independence
rs3729931	*RAF1*	T	1.42 (1.06-1.90)	0.021	1.73 (1.24-2.39)	0.001	0.326
rs45604736	*HRAS*	C	1.63 (1.11-2.40)	0.013	1.57 (1.06-2.32)	0.025	0.298
rs2283792	*MAPK1*	T	1.40 (1.03-1.91)	0.032	1.48 (1.08-2.03)	0.014	0.179
rs9610417	*MAPK1*	T	0.60 (0.38-0.93)	0.023	0.61 (0.38-0.98)	0.042	1.000

b) Tumour size

rsID	Gene	Minor allele	<5cm OR (95% CI)	*p*-value (unadjusted)	>5cm OR(95% CI)	*p*-value (unadjusted)	*p*-independence
rs3729931	*RAF1*	T	1.64 (1.19-2.26)	0.0024	1.47 (1.09-1.98)	0.012	0.75
rs45604736	*HRAS*	C	1.71 (1.16-2.51)	0.0070	1.50 (1.02-2.22)	0.039	0.51
rs2283792	*MAPK1*	T	1.24 (0.90-1.70)	0.19	1.63 (1.19-2.22)	0.0021	0.20
rs9610417	*MAPK1*	T	0.60 (0.37-0.95)	0.031	0.61 (0.39-0.96)	0.032	0.98

c) TNM Stage

rsID	Gene	Minor allele	TNM I or II OR (95% CI)	*p*-value (unadjusted)	TNM III or IV OR (95% CI)	*p*-value (unadjusted)	*p*-independence
rs3729931	*RAF1*	T	1.77 (1.23-2.55)	0.0021	1.45 (1.09-2.59)	0.010	0.46
rs45604736	*HRAS*	C	1.54 (1.00-2.37)	0.051	1.64 (1.14-2.37)	0.0080	0.13
rs2283792	*MAPK1*	T	1.45 (1.01-2.08)	0.043	1.41 (1.06-1.88)	0.017	0.96
rs9610417	*MAPK1*	T	0.52 (0.29-0.92)	0.024	0.66 (0.44-0.98)	0.041	0.38

Minor allele is the effect allele. OR (95%CI): Odds Ratio (95% Confidence Interval). OR estimation and *p*-value are unadjusted.
