# Supplementary material for: Polymorphisms in RAS/RAF/MEK/ERK Pathway Are Associated with Gastric Cancer
Source: Genes (Basel). 2018 Dec 28;10(1):20. doi: 10.3390/genes10010020 (PMC6356706; doi:10.3390/genes10010020)
Supplement: Supplementary file 1 [file genes-10-00020-s001.zip › table_S4.pdf]

Table S4. Proxy SNPs of associated variants and their functional annotation.

rs_ID	HGSV	Intron	Alleles (Major/minor)	MAF [1]	Distance (bases)	D'	r^2	Correlated alleles	RegulomeDB	enhancer histone marks	promoter histone marks	DNAse hypersensitive sites	ChIP-Seq (bound proteins)	eQTL
**rs3729931**														
rs3729931	NM_002880.3:c.1669-36C>T	15/16	(G/A)	0.47	0	1.00	1.00	G=G,A=A	1f	3	0	1	1	YES
**rs45604736**														
rs45604736	-	Upstream	(A/G)	0.12	0	1.00	1.00	A=A,G=G	2b	18	18	15	3	NO
rs117957959	-	Upstream	(C/T)	0.13	5825	1.00	0.97	A=C,G=T	5					NO
rs41258054	NM_176795.4:c.111+15G>A	2/5	(C/T)	0.14	-2280	1.00	0.88	A=C,G=T	5					NO
rs146436269	-	Upstream	(T/G)	0.14	6905	1.00	0.86	A=T,G=G	6					NO
**rs2283792**														
rs2283792	NM_002745.4:c.857-3854A>C	6/8	(T/G)	0.38	0	1.00	1.00	T=T,G=G	4					YES
rs11267198	NM_002745.4:c.120-2529_120-2528	1/8	(-/CATTAAAAAGAAA)	0.38	33538	1.00	0.99	T=-,G=CATTAAAAAGAAA	7					NO
rs11704205	NM_002745.4:c.610-2156A>G	4/8	(T/C)	0.38	14128	1.00	0.99	T=T,G=C	7					YES
rs13943	NM_002745.4:c.*2337G>C	3' UTR	(C/G)	0.38	-14923	1.00	0.99	T=C,G=G	5					YES
rs2266967	NM_002745.4:c.492+3350G>T	3/8	(C/A)	0.38	25664	1.00	0.99	T=C,G=A	5					YES
rs4478016	NM_002745.4:c.609+4570A>G	4/8	(T/C)	0.38	17606	1.00	0.99	T=T,G=C	7					YES
rs5749806	intergenic	intergenic	(C/A)	0.38	-24507	1.00	0.99	T=C,G=A	5					YES
rs5749986	NM_002745.4:c.492+2478A>G	3/8	(T/C)	0.38	26536	1.00	0.99	T=T,G=C	5					YES
rs5999521	NM_002745.4:c.610-3302T>C	4/8	(A/G)	0.38	15274	1.00	0.99	T=A,G=G	2b	9	1	5	14	YES
rs5999550	NM_002745.4:c.609+2015G>C	4/8	(C/G)	0.38	20161	1.00	0.99	T=C,G=G	4					YES
rs8136590	NM_002745.4:c.609+4158A>G	4/8	(T/C)	0.38	18018	1.00	0.99	T=T,G=C	7					YES
rs3810609	downstream	downstream	(C/T)	0.38	-18323	0.99	0.99	T=C,G=T	4					YES
rs3810610	NM_002745.4:c.*3041A>G	3' UTR	(T/C)	0.38	-15627	0.99	0.99	T=T,G=C	4					YES
rs1892846	NM_002745.4:c.120-24344T>G	1/8	(A/C)	0.38	55354	1.00	0.99	T=A,G=C	5					YES
rs1892848	NM_002745.4:c.120-24427A>G	1/8	(T/C)	0.38	55437	1.00	0.99	T=T,G=C	5					YES
rs2283794	NM_002745.4:c.119+9142A>T	1/8	(T/C)	0.38	81344	1.00	0.99	T=T,G=C	4					YES
rs5749937	NM_002745.4:c.856+2410G>A	6/8	(C/T)	0.38	9011	1.00	0.99	T=C,G=T	6					YES

rs_ID	HGSV	Intron	Alleles (Major/minor)	MAF [1]	Distance (bases)	D'	r^2	Correlated alleles	RegulomeDB	enhancer histone marks	promoter histone marks	DNAse hypersensitive sites	ChIP-Seq (bound proteins)	eQTL
rs5999750	NM_002745.4:c.120-25874A>G	1/8	(T/C)	0.38	56884	1.00	0.99	T=T,G=C	6					YES
rs5999823	NM_002745.4:c.119+10735G>T	1/8	(C/A)	0.38	79751	1.00	0.99	T=C,G=A	6					NO
rs6518986	NM_002745.4:c.120-23212A>G	1/8	(T/C)	0.38	54222	1.00	0.99	T=T,G=C	5					YES
rs5750113	NM_002745.4:c.119+1538G>A	1/8	(C/T)	0.38	88949	1.00	0.98	T=C,G=T	4					YES
rs5999515	NM_002745.4:c.610-2004A>G	4/8	(T/C)	0.38	13976	1.00	0.97	T=T,G=C	6					YES
rs8141851	NM_002745.4:c.120-4315T>C	1/8	(A/G)	0.38	35325	1.00	0.97	T=A,G=G	5					YES
rs8136867	NM_002745.4:c.119+16818C>T	1/8	(G/A)	0.37	73668	1.00	0.93	T=G,G=A	5					YES
rs5755654	NM_002745.4:c.119+29134T>C	1/8	(A/G)	0.37	61352	1.00	0.93	T=A,G=G	3a					YES
rs5755099	NM_002745.4:c.967-1431T>G	7/8	(A/C)	0.40	-6085	1.00	0.92	T=A,G=C	5					YES
rs6928	NM_002745.4:c.*3535G>C	3' UTR	(C/G)	0.40	-16121	0.99	0.91	T=C,G=G	4					YES
rs113244990	NM_002745.4:c.119+27940_119+279	1/8	(-/AAAC)	0.36	62542	1.00	0.91	T=-,G=AAAC	5					NO
rs11440648	intergenic	intergenic	(-/T)	0.39	99755	0.96	0.91	T=-,G=T	4					YES
rs9607347	intergenic	intergenic	(T/C)	0.39	96323	0.96	0.91	T=T,G=C	7					YES
rs5754941	intergenic	intergenic	(C/G)	0.36	-29130	0.99	0.87	T=C,G=G	7					YES
rs743409	NM_002745.4:c.857-1944T>C	6/8	(A/G)	0.34	-1910	1.00	0.84	T=A,G=G	4					YES
rs3788332	NM_002745.4:c.120-9432C>T	1/8	(G/A)	0.43	40442	0.99	0.83	T=G,G=A	2b	21	10	19	12	YES
rs4516540	NM_002745.4:c.120-6111T>C	1/8	(A/G)	0.43	37121	0.99	0.83	T=A,G=G	5					YES
rs5749998	NM_002745.4:c.120-239T>A	1/8	(A/T)	0.43	31249	0.99	0.83	T=A,G=T	5					YES
rs9607287	NM_002745.4:c.120-1290T>C	1/8	(A/G)	0.43	32300	0.99	0.83	T=A,G=G	7					YES
rs2006893	NM_002745.4:c.857-1407C>T	6/8	(G/A)	0.43	-2447	1.00	0.83	T=G,G=A	4					YES
**rs9610417**														
rs9610417	NM_002745.4:c.119+21641G>A	1/8	(C/T)	0.18	0	1.00	1.00	C=C,T=T	5					YES
rs9610386	NM_002745.4:c.120-28740G>A	1/8	(C/T)	0.18	-9095	1.00	1.00	C=C,T=T	6					YES
rs2070505	NM_002745.4:c.120-122C>G	1/8	(G/C)	0.18	-37713	1.00	0.99	C=G,T=C	4					YES
rs9607272	NM_002745.4:c.856+6148A>C	6/8	(T/G)	0.18	-63572	1.00	0.99	C=T,T=G	1f	2	0	6	1	YES
rs9610496	NM_002745.4:c.119+2163T>A	1/8	(A/T)	0.18	19479	1.00	0.99	C=A,T=T	2b	14	18	48	14	YES

rs_ID	HGSV	Intron	Alleles (Major/minor)	MAF [1]	Distance (bases)	D’	r^2	Correlated alleles	RegulomeDB	enhancer histone marks	promoter histone marks	DNAse hypersensitive sites	ChIP-Seq (bound proteins)	eQTL
rs3810608	-	Downstream	(G/A)	0.18	-87176	1.00	0.99	C=G,T=A	4					YES
rs61763392	NM_002745.4:c.856+651A>G	6/8	(T/C)	0.18	-58075	1.00	0.98	C=T,T=C	6					YES
rs3810606	-	Downstream	(A/G)	0.18	-88662	0.99	0.98	C=A,T=G	4					YES
rs6518951	NM_002745.4:c.966+589A>G	7/8	(T/C)	0.18	-73397	0.99	0.98	C=T,T=C	5					YES
rs111665812	NM_002745.4:c.609+4133G>A	4/8	(C/T)	0.19	-50802	1.00	0.94	C=C,T=T	7					YES
rs113709132	NM_002745.4:c.120-2714G>A	1/8	(C/T)	0.19	-35121	1.00	0.94	C=C,T=T	5					YES
rs28803281	NM_002745.4:c.609+4777G>A	4/8	(C/T)	0.19	-51446	1.00	0.94	C=C,T=T	7					NO
rs75681740	NM_002745.4:c.119+4656T>A	1/8	(A/T)	0.19	16986	1.00	0.94	C=A,T=T	5					NO
rs9610470	NM_002745.4:c.119+12472A>G	1/8	(T/C)	0.19	9169	1.00	0.94	C=T,T=C	1f	12	0	2	0	YES
rs143334716	NM_002745.4:c.119+19191dup	1/8	(-/C)	0.19	2449	1.00	0.93	C=-,T=C	3a					YES
rs62235142	NM_002745.4:c.610-1966C>T	4/8	(G/C)	0.19	-54907	1.00	0.93	C=G,T=C	7					YES
rs62235144	NM_002745.4:c.120-14383G>T	1/8	(C/A)	0.19	-23452	1.00	0.93	C=C,T=A	7					YES
rs9610446	NM_002745.4:c.119+18003T>G	1/8	(A/C)	0.19	3638	1.00	0.93	C=A,T=C	5					NO
rs9610487	NM_002745.4:c.119+7642A>G	1/8	(T/C)	0.19	13999	1.00	0.93	C=T,T=C	1d	13	1	8	1	YES
rs9610504	NM_002745.4:c.119+82C>T	1/8	(G/A)	0.18	21560	0.97	0.90	C=G,T=A	2b	0	24	38	16	YES
rs71735050	-	Downstream	(TGTT/-)	0.18	26741	0.94	0.87	C=TGTT,T=-	-					YES

HGSV: Human Genome Variation Society nomenclature, MAF: minor allele frequency, eQTL: expression Quantitative Trait Loci, ChIP-Seq: Chromatin Immunoprecipitation Sequencing.

[1]: 1000 Genomes Project Phase 3 (Version5), Ad Mixed American (AMR) population.

RS_Number	HGSV	Intron	Alleles (Major/minor)	MAF	Distance (bases)	D’	R2	Correlated_Alleles	RegulomeDB	enhancer histone marks	promoter histone marks	DNAse hypersensitive sites	ChIPseq	eQTL
rs45604736			(A/G)	0.12	0	1	1.00	A=A,G=G	2b	18	18	15	EBF1, MTA3, POLR2A	NO
rs117957959			(C/T)	0.13	5825	1	0.97	A=C,G=T	5					NO
rs41258054	NM_176795.4:c.111+15G>A	2/5	(C/T)	0.14	-2280	1	0.88	A=C,G=T	5					NO
rs146436269			(T/G)	0.14	6905	1	0.86	A=T,G=G	6					NO

RS_Number	HGSV	Intron	Alleles (Major/minor)	MAF	Distance (bases)	D’	R2	Correlated_Alleles	RegulomeDB
rs2283792	NM_002745.4:c.857-3854A>C	6/8	(T/G)	0.385	0	1	1	T=T,G=G	4
rs11267198	NM_002745.4:c.120-2529_120-2528insTTTCTTTTTAATG	1/8	(-/CATTA	0.383	33538	1	0.994	T=-,G=CATTAAAAAGAAA	7
rs11704205	NM_002745.4:c.610-2156A>G	4/8	(T/C)	0.383	14128	1	0.994	T=T,G=C	7
rs13943	NM_002745.4:c.*2337G>C	3’ UTR	(C/G)	0.383	-14923	1	0.994	T=C,G=G	5
rs2266967	NM_002745.4:c.492+3350G>T	3/8	(C/A)	0.383	25664	1	0.994	T=C,G=A	5
rs4478016	NM_002745.4:c.609+4570A>G	4/8	(T/C)	0.383	17606	1	0.994	T=T,G=C	7
rs5749806	intergenic	intergenic	(C/A)	0.383	-24507	1	0.994	T=C,G=A	5
rs5749986	NM_002745.4:c.492+2478A>G	3/8	(T/C)	0.383	26536	1	0.994	T=T,G=C	5
**rs5999521**	NM_002745.4:c.610-3302T>C	4/8	(A/G)	0.383	15274	1	0.994	T=A,G=G	**2b**
rs5999550	NM_002745.4:c.609+2015G>C	4/8	(C/G)	0.383	20161	1	0.994	T=C,G=G	4
rs8136590	NM_002745.4:c.609+4158A>G	4/8	(T/C)	0.383	18018	1	0.994	T=T,G=C	7
rs3810609	downstream	downstream	(C/T)	0.385	-18323	0.994	0.988	T=C,G=T	4
rs3810610	NM_002745.4:c.*3041A>G	3’ UTR	(T/C)	0.385	-15627	0.994	0.988	T=T,G=C	4
rs1892846	NM_002745.4:c.120-24344T>G	1/8	(A/C)	0.382	55354	1	0.988	T=A,G=C	5
rs1892848	NM_002745.4:c.120-24427A>G	1/8	(T/C)	0.382	55437	1	0.988	T=T,G=C	5
rs2283794	NM_002745.4:c.119+9142A>T	1/8	(T/C)	0.382	81344	1	0.988	T=T,G=C	4
rs5749937	NM_002745.4:c.856+2410G>A	6/8	(C/T)	0.382	9011	1	0.988	T=C,G=T	6
rs5999750	NM_002745.4:c.120-25874A>G	1/8	(T/C)	0.382	56884	1	0.988	T=T,G=C	6
rs5999823	NM_002745.4:c.119+10735G>T	1/8	(C/A)	0.382	79751	1	0.988	T=C,G=A	6
rs6518986	NM_002745.4:c.120-23212A>G	1/8	(T/C)	0.382	54222	1	0.988	T=T,G=C	5
rs5750113	NM_002745.4:c.119+1538G>A	1/8	(C/T)	0.38	88949	1	0.982	T=C,G=T	4
rs5999515	NM_002745.4:c.610-2004A>G	4/8	(T/C)	0.378	13976	1	0.97	T=T,G=C	6

rs8141851	NM_002745.4:c.120-4315T>C	1/8	(A/G)	0.378	35325	1	0.97	T=A,G=G	5
rs8136867	NM_002745.4:c.119+16818C>T	1/8	(G/A)	0.369	73668	1	0.935	T=G,G=A	5
**rs5755654**	NM_002745.4:c.119+29134T>C	1/8	(A/G)	0.367	61352	1	0.929	T=A,G=G	**3a**
rs5755099	NM_002745.4:c.967-1431T>G	7/8	(A/C)	0.404	-6085	1	0.925	T=A,G=C	5
rs6928	NM_002745.4:c.*3535G>C	3' UTR	(C/G)	0.404	-16121	0.994	0.913	T=C,G=G	4
rs113244990	NM_002745.4:c.119+27940_119+27943dup	1/8	(-/AAAC)	0.362	62542	1	0.906	T=-,G=AAAC	5
rs11440648	intergenic	intergenic	(-/T)	0.391	99755	0.963	0.905	T=-,G=T	4
rs9607347	intergenic	intergenic	(T/C)	0.391	96323	0.963	0.905	T=T,G=C	7
rs5754941	intergenic	intergenic	(C/G)	0.357	-29130	0.987	0.866	T=C,G=G	7
rs743409	NM_002745.4:c.857-1944T>C	6/8	(A/G)	0.344	-1910	1	0.84	T=A,G=G	4
**rs3788332**	NM_002745.4:c.120-9432C>T	1/8	(G/A)	0.425	40442	0.994	0.835	T=G,G=A	**2b**
rs4516540	NM_002745.4:c.120-6111T>C	1/8	(A/G)	0.425	37121	0.994	0.835	T=A,G=G	5
rs5749998	NM_002745.4:c.120-239T>A	1/8	(A/T)	0.425	31249	0.994	0.835	T=A,G=T	5
rs9607287	NM_002745.4:c.120-1290T>C	1/8	(A/G)	0.425	32300	0.994	0.835	T=A,G=G	7
rs2006893	NM_002745.4:c.857-1407C>T	6/8	(G/A)	0.431	-2447	1	0.826	T=G,G=A	4

enhancer histone marks	promoter histone marks	DNAse hypersensitive sites	ChIPseq	eQTL	polymiRST
				YES	
				#N/A	
				YES	
				YES	X
				YES	
				YES	
				YES	
				YES	
**9**	1	5	14	YES	
				YES	
				YES	
				YES	
				YES	X
				YES	
				YES	
				YES	
				YES	
				YES	
				#N/A	
				YES	
				YES	
				YES	

				YES	
				YES	
**14**	0	0		YES	
				YES	
				YES	
				#N/A	
				YES	
				YES	
				YES	
				YES	
**21**	10	19	12	YES	
				YES	
				YES	
				YES	
				YES	

RS_Number	HGSV	Intron	Alleles (Major/minor)	MAF	Distance (bases)	D’	R2	Correlated_Alleles	RegulomeDB
rs3729931	NM_002880.3:c.1669-36C>T	15/16	G/A	0.4683	0	1	1	G=G,A=A	1f

enhancer histone marks	promoter histone marks	DNAse hypersensitive sites	ChIPseq	eQTL
3	0	1	1	YES
